# Machine Outputs Must Be Checked

**DOI:** 10.1007/s00062-021-01012-6

**Published:** 2021-04-09

**Authors:** Elias Kellner, Horst Urbach

**Affiliations:** 1 Dept. of Radiology, Medical Physics, Medical Center, University of Freiburg, Freiburg, Germany; 2 Dept. of Neuroradiology, Medical Center, University of Freiburg, Freiburg, Germany

We read with interest the multicenter manuscript of Psychogios et al. in which they reported on the comparison of infarct core and tissue at risk maps generated by four different vendors as well as visual Cerebral Blood Volume—Alberta Stroke Program Early CT Score (CBV-ASPECTS) and visually assessed collateral scores [1]. They related the maps of 182 patients undergoing mechanical thrombectomy (MT) and receiving a TICI 2b, 2c or III reperfusion to the clinical outcome assessed with the modified Rankin score (mRS) and the functional disability defined as mRS > 2. They calculated mean differences between RAPID (iSchemaView Inc, Menlo Parc, CA, USA) and other software packages and illustrated them with Bland-Altman plots. They concluded that the infarct core defined by the RAPID software correlates best with the clinical outcome whilst VEOcore (VEObrain GmbH, Freiburg, Germany) and syngo.via (Siemens Healthineers AG, Erlangen, Germany) overestimate the infarct core and Olea (OLEA medical Inc., La Ciotat, France) underestimates it [1].

The message is clear but can we trust it? In the manuscript the authors clearly state that out of 215 cases 33 cases have been excluded from the final analysis due to “… technical failure of at least 1 perfusion software”; however, if we take a look at the Bland-Altman plot of RAPID-VEOcore (only available in the Supplemental Material) there is a striking outlier in the infarct core volume difference of around −2131 mL (which is distinctly larger than an entire brain). This outlier leads to a massive bias in the statistics: it can be estimated that without the outlier the true mean difference between RAPID and VEOcore is in a very good agreement range of −1.5 mL instead of the −13.4 mL reported in the manuscript.

Thus, a single nonplausible outlier caused a significant difference and led to an erroneous conclusion that the VEOcore software is inappropriate for treatment decisions beyond the 6h window [1].

**Fig. 1:**
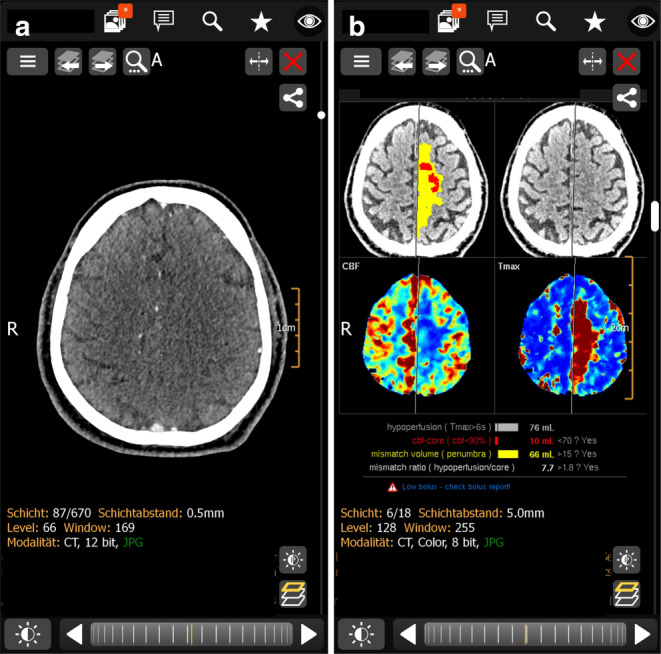
A 50-year-old man with right-sided hemiplegia and nonfluent aphasia since 1h was admitted in the evening. NIHSS was 17. The left-sided anterior cerebral artery occlusion was difficult to see on CT-Angiography (**a**, *left image*) using a mobile phone, but readily displayed on CT-Perfusion maps and the corresponding hypoperfusion- and core segmentation (**b**, *right image*)

Besides this inconsistency in the manuscript—which should be addressed in an erratum—we would further like to point out another general limitation of the work, which in our view leads to an increased distraction from the true value of CTP in acute stroke management in our community:

Postprocessing of CTP has two major goals: 1) to display the hypoperfused tissue and 2) to estimate how much of this tissue is already infarcted (infarct core). The only way to prove whether the infarct core is correctly identified on CTP is to compare it with a posttreatment MRI in recanalized patients and to calculate, for example, dice indices. To relate volume measurements to the clinical outcome is a too simplistic approach. We do not deny the weaknesses of CTP, however, the most accepted infarct core definition is tissue with a relative CBF < 30% compared to the contralateral hemisphere [2]. This threshold derived from the data of 103 patients in which DWI-MRI was acquired shortly after CTP [3] is implemented in the RAPID and VEOcore, but not in the original syngo.via software, for example [4]. In the meantime, apparently, the syngo.via software has also been adopted the < 30% rCBF value [5, 6]. Methodologically, issues such as different vulnerability of gray and white matter, order and timing of CTA and CTP, bolus interference of CTP and CTA, differences in CBF in gray and white matter (especially in patients with small vessel disease) must be considered when interpreting infarct core maps [7, 8]. CTP as well as the temporal thresholds applied in DAWN and DEFUSE 3 have empirically been chosen and are now required to indicate mechanical thrombectomy beyond the 6h window. Nevertheless, we use CTP in more situations. In medium and distal vessel occlusions it displays the hypoperfused tissue better than CTA (Fig. 1) and especially outside working hours it allows a rapid communication within a stroke network via mobile devices.

In conclusion, we appreciate a critical debate on the use of CTP using different postprocessing tools but such comparisons should be performed thoroughly and based on valid data.
